# Analyses of familial chylomicronemia syndrome in Pereira, Colombia 2010–2020: a cross-sectional study

**DOI:** 10.1186/s12944-022-01768-x

**Published:** 2023-03-28

**Authors:** Franklin Hanna Rodriguez, Jorge Mario Estrada, Henry Mauricio Arenas Quintero, Juan Patricio Nogueira, Gloria Liliana Porras-Hurtado

**Affiliations:** 1 International Center Research In Health Comfamiliar, Comfamiliar Risaralda, Pereira, Risaralda, Colombia; 2 Department of Endocrinology, Comfamiliar Risaralda, Pereira, Risaralda, Colombia; 3 Adjunct researcher of CONICET, National University of Formosa, Formosa, Argentina

**Keywords:** Familial hyperchylomicronemia syndrome, Multifactorial chylomicronemia, Hypertriglyceridemia, Moulin FCS score

## Abstract

**Background and aim:**

Familial chylomicronemia syndrome (FCS) is a rare autosomal recessive metabolic disorder caused by mutations in genes involved in chylomicron metabolism. On the other hand, multifactorial chylomicronemia syndrome (MCS) is a polygenic disorder and the most frequent cause of chylomicronemia, which results from the presence of multiple genetic variants related to chylomicron metabolism, in addition to secondary factors. Indeed, the genetic determinants that predispose to MCS are the presence of a heterozygous rare variant or an accumulation of several SNPs (oligo/polygenic). However, their clinical, paraclinical, and molecular features are not well established in our country. The objective of this study was to describe the development and results of a screening program for severe hypertriglyceridemia in Colombia.

**Methods:**

A cross-sectional study was performed. All patients aged >18 years with triglyceride levels ≥500 mg/dL from 2010 to 2020 were included. The program was developed in three stages: 1. Review of electronic records and identification of suspected cases based on laboratory findings (triglyceride levels ≥500 mg/dL); 2. Identification of suspected cases based on laboratory findings that also allowed us to exclude secondary factors; 3. Patients with FCS scores <8 were excluded. The remaining patients underwent molecular analysis.

**Results:**

In total, we categorized 2415 patients as suspected clinical cases with a mean age of 53 years, of which 68% corresponded to male patients. The mean triglyceride levels were 705.37 mg/dL (standard deviation [SD] 335.9 mg/dL). After applying the FCS score, 2.4% (n = 18) of patients met the probable case definition and underwent a molecular test. Additionally, 7 patients had unique variants in the *APOA5* gene (c.694 T > C; p. Ser232Pro) or in the *GPIHBP1* gene (c.523G > C; p. Gly175Arg), for an apparent prevalence of familial chylomicronemia in the consulting population of 0.41 per 1.000 patients with severe HTG measurement. No previously reported pathogenic variants were detected.

**Conclusion:**

This study describes a screening program for the detection of severe hypertriglyceridemia. Although we identified seven patients as carriers of a variant in the *APOA5* gene, we diagnosed only one patient with FCS. We believe that more programs of these characteristics should be developed in our region, given the importance of early detection of this metabolic disorder.

## Introduction

Hypertriglyceridemia (HTG) is a common medical condition associated with increased very-low-density lipoprotein (VLDL) and chylomicron levels, and in certain cases, it is associated with increased cardiovascular risk and pancreatitis [1, 2]. Based on recent genetic data, the disorder has been redefined into two types: severe HTG with triglyceride (TG) levels greater than 10 mmol/L (>880 mg/dL) or > 500 mg/dL, which is more likely to have a monogenic cause, and mild-to-moderate HTG with TG levels between 2–10 mmol/L (150–880 mg/dL) or 150–500 mg/dL [3, 4]. Severe HTG can be produced by primary causes associated with genetic disorders in the metabolism of lipids and by secondary causes, such as alcoholism, smoking, biliary disease, and uncontrolled diabetes [5].

There are two common causes of severe hypertriglyceridemia that we approach in this study. First, familial chylomicronemia syndrome (FCS) is an inherited autosomal recessive disease caused by mutations in the lipoprotein lipase (*LPL*) gene or, more rarely, by loss-of-function mutations in apolipoprotein C-II (*APOC2*), apolipoprotein A-V *(APOA5*), glycosyl-phosphatidylinositol anchored high-density lipoprotein-binding protein 1 (*GPIHBP1*) and lipase maturation factor 1 (*LMF1*) genes encoding proteins related to its modulation [6]. FCS is a disorder of lipid metabolism, especially chylomicrons, in which TGs are severely increased.

It is estimated that there could be approximately 3000 to 5000 patients worldwide with FCS [7]. At the general level, prevalence rates of 1–2 cases per 1000,000 inhabitants have been reported [8], and in some populations with a founder effect, prevalence rates of 1 case per 10,000 inhabitants can be found [9].

Clinically, this condition is characterized by recurrent abdominal pain, hepatomegaly, splenomegaly, eruptive xanthomas as a cutaneous manifestation, decreased levels of low-density lipoprotein (LDL) and low body mass index (BMI) and, most importantly, inflammatory processes at the pancreas level [10, 11].

When acutely present, they require specialized management at the clinical level and frequently in the intensive care unit due to the risk of lethal complications [12]. Pancreatic inflammatory processes can also occur as recurrent and chronic presentations, such as chronic pancreatitis or recurrent abdominal pain [12]. These chronic inflammatory states also lead to progressive and irreversible damage with fibrosis, obstruction, and glandular atrophy, causing exocrine and endocrine pancreatic insufficiency, as well as complications such as pseudocysts, abscesses, biliary stenosis, duodenal obstruction, diabetes, and an increased risk of pancreatic cancer [12]. Despite the severe and disabling clinical manifestations, the biomarker that draws the most attention to the suspicion of this disease is the severely elevated TG levels and its difficult management [13]. A recent meta-analysis has shown the rates of complications and mortality for acute pancreatitis (AP), establishing that they were significantly increased in patients with TGs >5.6 mM (>500 mg/dL) or > 11.3 mM (>1000 mg/dL) versus <5.6 mM (<500 mg/dL) or < 11.3 mM (<1000 mg/dL), respectively [14].

On the other hand, multifactorial chylomicronemia syndrome (MCS) is a polygenic disorder and the most frequent cause of CS that results from the presence of multiple genetic variants that include both rare heterozygous variants in the *LPL*, *APOC2*, *APOA5*, apolipoprotein B (APOB), glucokinase regulatory protein (*GCKR*), *LMF1*, *GPIHBP1*, and cyclic AMP-responsive element-binding protein 3-like protein 3 (*CREB3L3*) genes and more frequent variants with small effects in ~40 genes [15]. It is triggered by the presence of secondary factors such as a diet rich in fats and simple sugars, obesity, hypertension, alcohol intake, and uncontrolled diabetes. Indeed, the genetic determinants that predispose to MCS are the presence of a heterozygous rare variant or an accumulation of several SNPs (oligo/polygenic). In patients with MCS, chylomicronemia typically occurs later in life. It has been estimated that this chylomicronemia can be found in 1:600 adults, but FCS patients represent only 5% of these individuals [16]. Clinically, high serum TG levels and chylomicronemia are also characteristic of MCS, along with a similar presentation to FCS but with a different underlying etiology. Comparable with FCS, patients with this disorder can have fasting TG levels ≥880 mg/dL as well, putting them at an increased risk for AP [17].

This condition is underrecognized, with the majority of FCS patients being diagnosed after age 20, often after consulting several physicians [12]. Moreover, severe HTG can be observed in other metabolic conditions, including type III dysbetalipoproteinemia, familial combined hyperlipidemia, and familial hypertriglyceridemia [11]. Suspicion of MCS is found with permanently elevated TG levels ≥880 mg/dL and secondary causes. Other primary causes are ruled out throughout the study of the patients [12, 13, 18].

To date, only 166 patients with familial chylomicronemia have been diagnosed and confirmed by molecular genetic studies in Colombia due to limitations in patients’ access to health services and specialized laboratories in the country [19].

Considering the major clinical relevance of early identification of these patients and from a cost-effective point of view, some experts have proposed that it may be useful to have a diagnostic algorithm for FCS and MCS [20]. Regarding the information mentioned above, the objective of this study was to describe the clinical and paraclinical characteristics of patients with severe HTG results from a screening program for severe hypertriglyceridemia in Colombia.

## Materials and methods

We used a cross-sectional descriptive study that was hospital-based and carried out in stages. It was based on the use of a diagnostic screening test and a gold standard for verification restricted to only positive patients, following the methodology proposed by Shrout PE et al. and Mcnamee R. for prevalence studies in rare/orphan diseases [21, 22].

Three different case definitions were constructed according to three phases in the selection of patients in a progressive manner, as follows:

1. In the first stage, suspected cases, based on laboratory findings, were defined as patients with triglyceride levels ≥500 mg/dL.
2. In the second stage, all suspected cases were determined based on laboratory findings with TG levels, LDL, cholesterol, HDL, and APOB that also allowed us to exclude those with secondary factors.
3. In the third stage, patients with FCS scores <8 were excluded.

APOB and LDL levels were used to exclude mixed dyslipidemia. The molecular analysis was performed by sequencing the coding region of the genome (exome >20,000 genes) with a coverage greater than 98% and a minimum depth of 20X. From these data, the sequences of the *APOA5*, *APOC2*, *GPIHBP1*, *LMF1*, and *LPL* genes were analyzed, thus covering all the pathogenic variants of these genes in exonic or splicing regions (at least 20 bp), insertions, and small deletions, with aligned and filtered sequences meeting quality criteria with analysis performed for the hg19 assembly of the human genome.

The inclusion criteria involved patients over 18 years of age of either sex with triglyceride levels >500 mg/dL who consulted a fourth-level center in Colombia during the period from 2010 to 2020.

The information was collected in two stages: first, it was carried out utilizing a retrospective active search with the use of Structured Query Language (SQL) in institutional laboratory databases, obtaining the complete results of TGs processed institutionally, of which lower values ​​were filtered with age criteria greater than 18 years and triglyceride levels greater than 500 mg/dL. This triglyceride cutoff point of 500 mg/dL was chosen for this program because it was the definition used by the lipid clinic of our institution when the protocol was developed. Subsequently, a review of the literature was carried out to identify diagnostic conditions and pharmacological treatments that were related to the increase in TGs. In terms of diagnoses, they were located using the SQL queries and ICD-10 identified as confirmed and prescribed medications, in addition to obtaining the latest lipid profile results stored in the medical record.

In the second stage, patients with secondary factors for HTG were excluded. The remaining patients underwent a medical consultation to collect current information and to request a new APOB lipid profile and glycated hemoglobin (HbA1c).

In a third stage, a subsequent application of Moulin’s Familial Chylomicronemia Score was made, which is reported in the literature as a scoring system for the differential diagnosis of FCS [10, 23]. This tool allows assessment using a standardized score of values ​​of the current lipid profile, signs and symptoms, and response to lipid-lowering treatment. A total score was obtained, and all patients with a score lower than 8 were excluded. In addition, the remaining patients underwent diagnostic confirmation by molecular analysis.

The data were systematized through a clinical data questionnaire designed on the REDcap platform to guarantee standardization in the collection, as well as confidentiality and quality. The information extracted by SQL queries and registered in Redcap was processed in the R platform with the use of the Tidyverse package for data cleaning. Descriptive statistics were used to summarize categorical (relative frequencies) and continuous data (mean and standard deviation). The estimation of the prevalence was performed using 95% confidence intervals under the application of Bayesian methods according to the design used.

To evaluate the factors associated with patients classified as probable cases, comparisons were made for relevant variables as reported in the literature, such as sex, age, medical history (hypertension, diabetes and pancreatitis), and paraclinical results of HDL, LDL, and triglycerides. Parametric and nonparametric tests were used according to the assumptions of distribution and/or type of data.

Additionally, as part of the exploration and characterization of HTG, a multivariate logistic regression model was adjusted for the variable using triglyceride values greater than and equal to 1000 mg/dL, in which multiple other variables were included. (Table 1). The selection of variables was carried out using the Akaike information criteria, and the significance of the coefficients was evaluated using the Wald test. Adjusted ORs with 95% confidence intervals were obtained. All comparisons were evaluated for their significance with a type I error of 0,05.

**Table 1:** Clinical and demographic characteristics of patients categorized as suspected clinical cases

	*N* = 2415^a^
Sex	
F	783 (32%)
M	1632 (68%)
Age	52 (12)
Arterial Hypertension	771 (32%)
Diabetes mellitus	483 (20%)
Obesity	305 (13%)
Pancreatitis	15 (0.6%)
Classification	
FCS Score < 8	2357 (98%)
FCS Score > = 8	58 (2.4%)
BMI (kg/m^2)	27.6 (5.6)
HDL (mg/dL)	35 (9)
LDL (mg/dl)	104 (40)
Triglycerides (mg(dl)	705.37 (335.9)
TG Classification	
500–1000 mg/dl	2170 (90%)
1000–1500 mg/dl	188 (7.8%)
> 1500 mg/dl	57 (2.4%)

^a^n (%); Mean (SD)

## Results

**Fig. 1:**
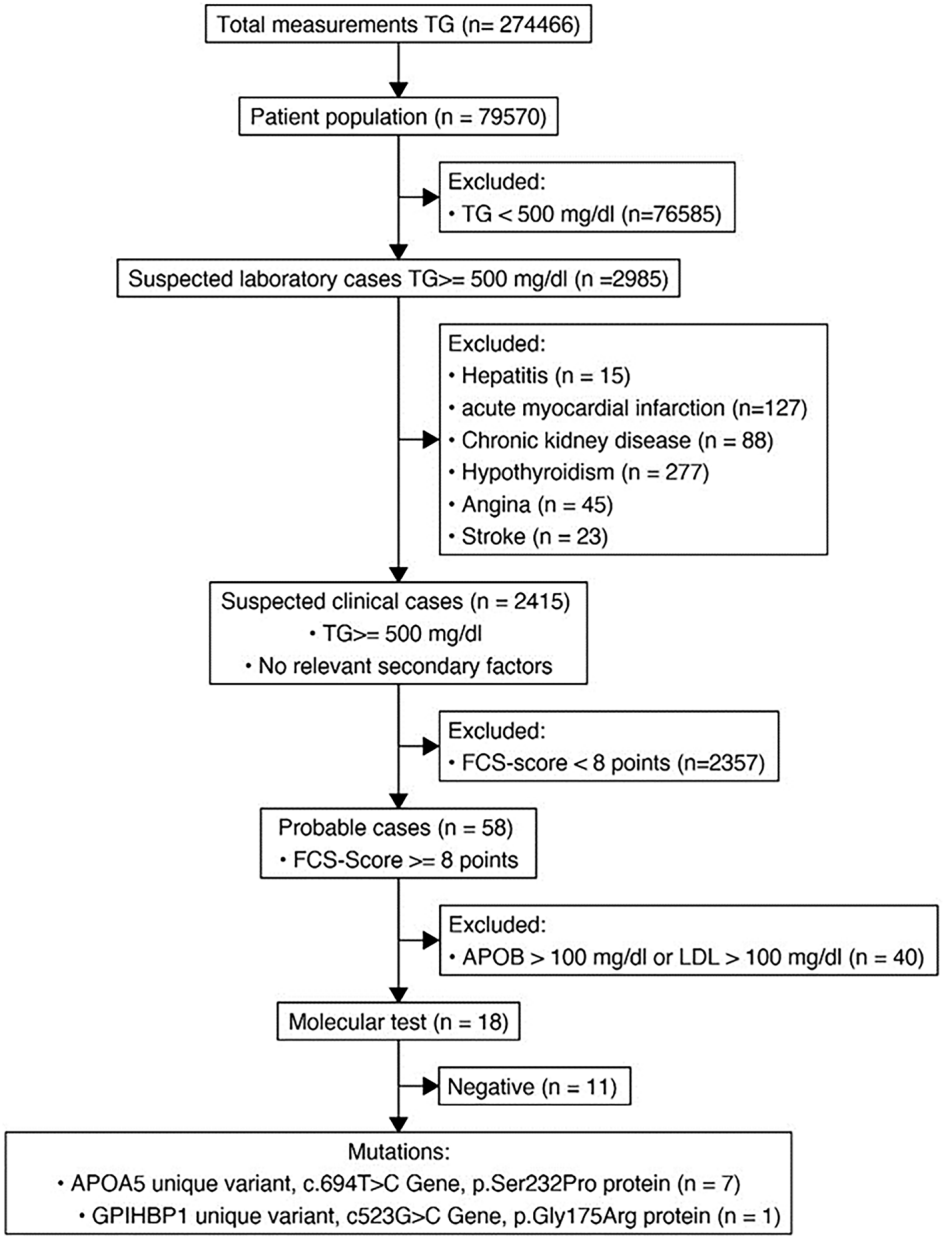
Patient selection diagram according to case definitions and study stage

274.466 TG measurements were identified in 79,570 patients. Individuals with other causes of HTG were excluded, and a total sample was obtained for analysis (Fig. 1). A total of 2.415 users had TG values greater than 500 mg/dL. Regarding distribution by sex, 1632 men corresponded to 68%, and 783 women corresponded to 32%. The prevalence of arterial hypertension in the evaluated group was 32%, and that of pancreatitis was 0.6%. A total of 2.4% of patients met the probable case definition. Other laboratory characteristics are reported in Table 1.

Fifty-eight patients with a score greater than 8 were identified, of which 40 patients were excluded due to elevated APOB and/or LDL levels (>100 mg/dL), suggesting mixed dyslipidemia. The remaining 18 patients underwent molecular testing, leading to 7 patients with unique variants in the *APOA5* gene (c.694 T > C; p. Ser232Pro) or the *GPIHBP1* gene (c.523G > C DNA, p. Gly175Arg), for a prevalence of familial chylomicronemia in the consulting population of 0.41 per 1000 patients with severe HTG.

In the adjusted regression model (Table 2), independent factors associated with severe HTG were male sex (OR 1.5, CI95% 1.1–2.1), diabetes mellitus (OR 1.5 CI95% 1.1–2.1) and an FCS score greater than 8 points (OR 1.3 CI95% 1.3–1.4). In contrast, obesity (OR 0.53 CI95% 0.3–0.9) was associated with a lower frequency among the group with severe HTG.

**Table 2:** Multiple logistic regression analysis for triglycerides greater than 1000 mg/dl

	OR^a^	95% CI^a^	*P* value
Sex (Male)	1.51	1.11, 2.08	0.011
Diabetes mellitus	1.55	1.12, 2.13	0.007
Obesity	0.53	0.31, 0.86	0.015
Score	1.34	1.26, 1.43	<0.001

^a^*OR* Odds Ratio Adjust, *CI* Confidence Interval

Six patients with (NM_052968.4) c.694 T > C, p. Ser232Pro protein, of uncertain significance in the *APOA5* gene associated with hyperlipoproteinemia type 1D (MIM # 615947)/autosomal recessive disease, were documented in carrier status. One patient had an additional variant in the *GPIHBP1* gene, c.523G > C DNA, p. Gly175Arg protein.

Table 3 documents the selection of 18 patients who underwent molecular genetic testing, 15 men and 3 women. From this selection, we found 7 samples with a mutation in the *APOA5* gene, c.694 T > C DNA, p. Ser232Pro protein, 4 men and 2 women; an additional mutation was documented in one sample: *GPIHBP1* gene, c.523G > C DNA, p. Gly175Arg protein. The identified variants were evaluated in the HGMD, ClinVar, LOVD, dbSNP, and gnomAD databases. For variants of uncertain significance (VUS), *in silico* prediction tools were used. Three patients, 2 men and 1 woman, with a history of recurrent acute pancreatitis were documented. Of these, 1 male patient had a diagnosis of well-controlled DM2 with an HbA1c of 6.42%. The second male patient was overweight with no other comorbidities, and the female patient had a diagnosis of obesity.

**Table 3:** List of patients with laboratory tests, pancreatitis and molecular panel results by sex

Sex	TG	APOB	HbA1C	LDL	HDL	BMI	Pancreatitis	Other pathologies	Molecular panel results
F	2988	89.7	5.06	71	35	29.75	NO	SAH, Overweight	APOA5 gene, c.694 T > C DNA, p.Ser232Pro protein, heterozygosity of uncertain significance
M	1808	57.2	5.43	45	29	30.22	NO	SAH, DM2, Overweight, Hipotiroidismo	No pathogenic variants have been identified.
M	1093	99.6	8.56	82	32	29.90	NO	DM2, SAH, AMI, obesity	No pathogenic variants have been identified.
M	1015	69.3	5.62	44	26	31.77	NO	Angina, obesity	APOA5 gene, c.694 T > C DNA, p.Ser232Pro protein, heterozygosity of uncertain significance; GPIHBP1 gene, c.523G > C DNA, p.Gly175Arg protein, heterozygosity of uncertain significance
M	2274	91.1	6.64	62	22	30.90	NO	DM2, obesity	No pathogenic variants have been identified.
M	314	69.5	6.41	56	21	39.89	NO	DM2, SAH, obesity	APOA5 gene, c.694 T > C DNA, p.Ser232Pro protein, heterozygosity of uncertain significance
M	1207	109	5.39	77	16	29.76	NO	DM2	No pathogenic variants have been identified.
M	2823	104	5.28	48	29	30.89	NO	DM2	No pathogenic variants have been identified.
M	907	86	5.05	78	29	24.80	NO	None	No pathogenic variants have been identified.
M	740	69	7.54	86	29	34.74	NO	DM2, SAH, Hypothyroidism, obesity	No pathogenic variants have been identified.
M	880	116	5.37	74	23	29.79	NO	None	No pathogenic variants have been identified.
M	1109	110	5.76	85	47	26.53	NO	SAH, Overweight	No pathogenic variants have been identified.
M	430	75	4.8	77	31	27.27	NO	NA	No pathogenic variants have been identified.
F	739	66	5.43	63	50	33.10	NO	Hypothyroidism, obesity	No pathogenic variants have been identified.
M	448	70	7.24	57	39	36.65	NO	DM2, SAH, Hypothyroidism, obesity	No pathogenic variants have been identified.
M	1333	139	5.37	43	21	28.41	NO	Overweight	APOA5 gene, c.694 T > C DNA, p.Ser232Pro protein, heterozygosity of uncertain significance
F	1628	NA	4.74	66.9	28	31.27	YES	obesity	APOA5 gene, c.694 T > C DNA, p.Ser232Pro protein, heterozygosity of uncertain significance
M	1984	NA	6.42	42.8	16	27.68	YES	DM	APOA5 gene, c.694 T > C DNA, p.Ser232Pro protein, heterozygosity of uncertain significance
M	524	77.7	5.75	50.2	24	–	YES	Overweight	APOA5 gene, c.694 T > C DNA, p.Ser232Pro protein, heterozygosity of uncertain significance

*SAH* systemic arterial hypertension, *DM* diabetes mellitus, *BMI* body mass index

## Discussion

We described a screening program for severe HTG in Colombia. Figure 1 shows that 40 patients with elevated APOB and/or LDL levels suggesting mixed dyslipidemia were excluded according to a multivariate analysis in two published works that have been to be the best predictors of FCS [17, 24]. On the other hand, patients with CVD were also excluded because in real life, most patients are treated with statins that alter lipid metabolism. It is worth mentioning that the samples obtained for this study were taken in the city of Pereira, Colombia, with 477,027 habitants. Therefore, it constitutes a representative sample for the Risaralda Region.

Although HTG has a frequent presentation in clinical practice, severe HTG has a prevalence of only 1.7% in the United States [25], and FCS represents 1–3% of severe HTGs [12]. The prevalence of severe HTG in the present study was 3% (95% credible interval: 2.9–3.2) (n = 2415), with FCS representing a lower prevalence of 0.062% (95% credible interval: 0.0000657–0.16) of those severe HTGs, most likely due to a different cutoff point. Furthermore, the vast majority of these patients have MCS. Although the clinical suspicion could be somewhat simple (when the TG levels are extremely high), the low prevalence of the disease and the lack of knowledge and awareness might lead many patients to remain underdiagnosed. Moreover, patients can remain misdiagnosed or be diagnosed in advanced stages when they have already had complications derived from the disease [10, 16, 20, 26–28].

Between 2011 and 2013, a higher prevalence of HTG was identified in Russia in men ranging from 40 to 49 years old (42.8%) and women from 60 to 69 years old (34.4%) [29]. In the present study performed in a fourth-level care center in Pereira, Colombia, in the period between 2010 and 2020, 64.19% of patients with HTG were men, with a larger proportion between 45 and 55 years old, and in comparison, 35.81% were women, with a higher proportion between 50 and 60 years old. This is in concordance with the higher proportion of men in the SIMETAP-HTG study, which showed a prevalence of 34,6% for men and 21,4% for women [23]. The clinical manifestations of HTG are similar to those of both MCS and FCS, with symptoms extending from eruptive xanthomas, lipemia retinalis, and hepatosplenomegaly to recurrent episodes of AP. In this study, we found recurrent AP in 3 patients with no other signs or symptoms related to CS.

It is estimated that up to 85% of patients with severe HTG of any etiology will develop AP at some point in their life [30]. According to Retterstøl, K., et al., the prevalence of a history of AP is 17% in patients with severe HTG [18]. AP as a consequence of HTG represents the third leading cause of pancreatitis after biliary and alcoholic etiologies. Therefore, severe HTG constitutes a therapeutic challenge due to the possibility of developing hyperlipidemic acute pancreatitis, especially when TG levels exceed 1000 mg/dL, as the risk increases up to 5% [31]. In our study, the prevalence of pancreatitis in patients with FCS score ≥ 8 was significantly higher (6.9%) compared to those with a score < 8 (0.5%) (Table 4). A recent meta-analysis showed that pancreatitis caused by HTG is associated with higher levels of severity, mortality, and hospitalizations in intensive care units than other etiologies [14].

**Table 4:** Comparative analysis for the identification of factors associated with a probable case of familial chylomicronemia

	Score < 8, *N* = 2357^1^	Score > = 8, *N* = 58^1^	*P* value^2^
Sex			0.053
F	771 (33)	12 (21)	
M	1586 (67)	46 (79)	
Age	52 (12)	50 (11)	0.3
Arterial Hypertension	760 (32)	11 (19)	0.032
Diabetes mellitus	461 (20)	22 (38)	<0.001
Obesity	303 (13)	2 (3.4)	0.033
Pancreatitis	11 (0.5)	4 (6.9)	<0.001
BMI (kg/m^2)	27.6 (5.3)	26.8 (7.4)	0.6
HDL (mg/dl)	35 (9)	29 (8)	<0.001
LDL (mg/dl)	105 (40)	76 (40)	<0.001
Triglycerides (mg(dl)	469 (357)	1033 (905)	<0.001

^1^n (%); Mean (SD)

^2^Pearson's Chi-squared test; Wilcoxon rank sum test; Fisher's exact test

Thong et al. found in a study that in a population with very severe HTG, 13.4% of patients with HTG-induced acute pancreatitis had dyslipidemia, 15.9% presented hypertension and 28% had type 2 diabetes mellitus [30]. In the present study, 49.28, 32.43, and 19.6% of patients were identified with dyslipidemia, hypertension, and DM2, respectively. In addition, a prevalence of obesity of 12.9% was found, a figure lower than the data observed in the Norwegian study, which was 41.6% [18]. On the other hand, Pedragosa et al. found that 10.2% of patients with very severe HTG presented some episode of pancreatitis, 54% of patients had hepatic steatosis, 4.2% had gallstones, 3.4% had ischemic heart disease, 2.3% had cerebrovascular disease and 2.7% had peripheral artery disease [32]. These findings suggest that although pancreatitis is a major complication, it is important to be aware of cardiovascular outcomes as well. Goldberg et al. proposed that patients with FCS are younger and less likely to have any of the aggravating factors for HTG than those with MCS but are more likely to develop pancreatitis, probably because of life-long, sustained chylomicronemia [33]. They are less likely to have cardiovascular disease than those with MCS because of the severe reduction in LPL activity, which reduces atherogenic chylomicron and VLDL remnant formation and accumulation that may occur in MCS [33].

FCS results from mutations in one or more genes involved in lipolysis or the removal of circulating chylomicrons. In approximately 30% of patients, it is not possible to identify a specific causal variant. In this study, the presence of a variant (c.694 T > C, p. Ser232Pro) in the *APOA5* gene (NM_052968.4), of uncertain significance associated with hyperlipoproteinemia-type disease 1D (MIM # 615947)/autosomal recessive in the carrier state was identified. When analyzing the clinical and paraclinical characteristics of these patients, 3 presented pancreatitis, overweight, or obesity with no other pathologies. It was confirmed through MLPA that 1 patient had an additional c. (?_50–1)_(161 + 1_162–1)dup duplication in the *APOA5* gene, which makes the patient a compound heterozygote, whereby it would be the only confirmed diagnosis for familial chylomicronemia within the cohort at clinical evaluation that only had pancreatitis of the typical clinical picture of chylomicronemia.

APOA5 is a lipoprotein secreted in the liver, with concentrations of 150–400 ng/mL in plasma, which corresponds to 10,000 times less than the secretion of APOA1. Its structure is composed of 4 exons that encode 366 amino acids, similar in 27% to APOA4 in humans, located on chromosome 11q23 [34]. This gene is one of those responsible for facilitating the metabolism of triglycerides through the hydrolysis of LPL [34]. Several gene changes have been found to cause increased cardiovascular risk and severe HTG [35]. VUS, identified as common according to databases, is frequent in patients in this region.

There is evidence for several genes that act in the modulation and homeostasis of triglycerides. One of them is the *GPIHBP1* gene, which encodes a glycoprotein of the lymphocyte antigen 6 family that is produced in the endothelium [36]. This glycoprotein is responsible for transporting LPL from the interstitium to the capillary lumen and subsequently to the capillary for LPL activation. Its structure consists of 4 exons and 228 amino acids with an N-terminal sequence followed by a C-terminal hydrophobic region similar to a glycosylphosphatidylinositol anchor [37].

One of the patients also had a variant in the *GPIHBP1* gene (c.523G > C, p. Gly175Arg), also of uncertain clinical significance, associated with hyperlipoproteinemia type 1D (MIM # 615947) plus the variant in *APOA5* already described in the carrier state for both genes, without a typical phenotype for familial chylomicronemia. Other variants in the same genes have been seen in other studies, such as in Singapore, where Loh et al. described a case report of a patient with a homozygous variant in *APOA5* and a heterozygous common variant in GPIHBP1 who presented with subarachnoid hemorrhage with lactescent appearance [38]. In addition, in Norway, Retterstøl et al. diagnosed 2 female patients with primary HTG and a history of recurrent AP who had a homozygous mutation in exons 3 and 4 of the GPIHBP1 gene, and Lin et al. found a homozygous mutation in the same gene in a patient with a history of recurrent AP without mutations in the *APOA5* gene [18, 36].

We only identified VUS in this study, making a diagnosis of FCS highly difficult to establish. We highlight the fact that these VUS were analyzed for cost-effectiveness in order to be able to characterize the clinical and biochemical parameters of the FCS, in which we compared the subgroups of score > 8 vs <8. Future studies should analyze enzymatic APOA5 levels to confirm the pathogenesis of this VUS.

Treatments for both FCS and MCS are almost different, constituting an important challenge for an adequate early diagnostic strategy. Most MCS patients have a good response to modifications in lifestyle, treatment of secondary factors, and triglyceride-lowering pharmacotherapies [16]. On the other hand, FCS patients have a poor response to triglyceride-lowering therapies such as fibrates, highlighting the need for other therapeutic strategies such as an extremely severe diet that restricts the consumption of long-chain fatty acids. Fortunately, there are recent studies with novel therapies such as inhibitors of apolipoprotein C-III under development to lower triglycerides in FCS subjects [16].

It is important to screen not only FCS patients but also MCS patients since those with some rare variants might be predisposed to worse phenotypes. Paquette et al. suggest genetic screening in patients with TGs ≥10 mmol/L to identify those at higher risk for AP [39]. However, this was not the purpose of this study. We aimed to describe a screening program for FCS with a stepwise approach, in which scores >8 or < 8 allowed us to analyze two subgroups of patients with FCS and MCS.

### Study strengths and limitations

The strengths of this study are the sample size, and the fact that this was the first study exploring familial chylomicronemia syndrome in this region, proved that the unique variant we found has some degree of association in pancreatitis or death. In regard with limitations, the VUS found in this study needs further confirmation by enzyme levels. In addition, data was extracted by text mining using regular expressions, which could have not detected some conditions due to the writing methods because of its natural language. This could have affected the frequencies of signs and symptoms that might not have been written in the clinic histories, resulting in a possible subregistry.

## Conclusions

In Colombia, the presence of the c.694 T > C; p. Ser232Pro unique variant of the *APOA5* gene has been confirmed in seven patients with severe HTG that, together with another heterozygous variant or alcohol, might result in pancreatitis or death. This pathology is infrequent but has a high impact on the quality of life and families of those who suffer it. Of the total number of evaluated patients, one patient with two mutations was identified as a requirement for establishing a diagnosis of FCS. Therefore, it is crucial to consider that a patient with severe HTG can undergo a molecular analysis to improve prognosis and outcomes.

We highlight the benefits of performing more studies directed to all populations. In this way, our study will contribute to the epidemiological knowledge and profile of patients with HTG.

## Data Availability

All data generated or analyzed during this study are included in this published article (and its supplementary information files).

## Abbreviations

HTG: Hypertriglyceridemia
VLDL: very-low-density lipoprotein
LDL: Low-density lipoprotein
BMI: Body mass index
TG: Triglyceride
FCS: Familial chylomicronemia syndrome
LPL: Lipoprotein lipase
AP: Acute pancreatitis
MCS: Multifactorial chylomicronemia syndrome
